# Diagnostic accuracy of magnetic resonance-guided prostate biopsy and template-guided transperineal saturation biopsy

**DOI:** 10.1097/MD.0000000000012495

**Published:** 2018-09-21

**Authors:** Yi Zhou, Zhien Zhou, Qianyue Li, Yinyan Xu, Hao Sun, Yu Xiao, Zhiyong Liang, Weigang Yan, Zhigang Ji, Hanzhong Li

**Affiliations:** a Department of Urology, Peking Union Medical College Hospital, Chinese Academy of Medical Sciences, Beijing; b Department of Urology, General Hospital of Xinjiang Production and Construction Corps, Urumqi; c Department of Radiology; d Department of Pathology, Peking Union Medical College Hospital, Chinese Academy of Medical Sciences, Beijing, People's Republic of China.

**Keywords:** biopsy, MRI, prostate, transperineal, distribution

## Abstract

To compare the accuracy of magnetic resonance-guided prostate biopsy (MR-GPB) and template-guided transperineal prostate saturation biopsy (TTPSB).

A total of 219 patients with elevated prostate-specific antigen, abnormal digital rectal examination or ultrasound findings were enrolled. All patients underwent multiparametric magnetic resonance image (mpMRI). Patients with a Prostate Imaging Reporting and Data System (PI-RADS) score of 3 to 5 underwent MR-GPB using 2 to 5 biopsy cores and then immediately underwent an 11-region TTPSB. Patients with a PI-RADS score of 1 to 2 underwent TTPSB alone. We compared the detection rates for any cancer, clinically significant prostate cancer (csPCA), and the spatial distribution of missed csPCA lesions.

Among the 219 cases, 66 (30.1%) had a PI-RADS score of 1 to 2 on mpMRI. The detection rate of TTPSB in these patients was 9.1% (6/66). In total, detection rates for any cancer and csPCA were 48.9% (107/219) and 42.9% (94/219), respectively. Detection rates for any cancer (TTPSB 87/219, 39.7%; MR-GPB76/219, 34.7%, *P = *.161) and csPCA (TTPSB 76/219, 34.7%; MR-GPB 72/219, 32.9%, *P = *.636) did not significantly differ between the 2 groups. The csPCA lesions missed by MR-GPB were most commonly located on the left (8.5%, 8/94) and right (9.6%, 9/94) sides of the urethra.

MR-GPB can reduce the rate of unnecessary prostate biopsies by approximately 30% and exhibits an efficacy comparable to TTPSB for the detection of any cancer and csPCA. Nevertheless, approximately 1/4 of csPCAs were missed by MR-GPB and were most commonly located on both sides of the urethra.

## 1 Introduction

Treatment decisions in prostate diseases are largely based on the results of biopsies. Currently, 12-core systemic transrectal ultrasound (TRUS)-guided biopsy is most commonly used in practice; however, its use has been challenged due to a relatively high false negative rate, low detection rate in the anterior and apex zones, and underestimation of the risk stratification of prostate cancer.^[1]^ Alternatively, transperineal prostate biopsy, especially template-guided transperineal prostate saturation biopsy (TTPSB), acquires saturated biopsies of the whole prostate and is more sensitive for detection of cancer lesions in the anterior and apex zones.^[2]^ Although TTPSB can provide a more accurate Gleason score,^[3]^ concerns with its use have also been expressed, such as over-diagnosis.

In recent years, magnetic resonance-guided prostate biopsy (MR-GPB) has been commonly applied in the evaluation of prostate cancer. Many studies have concluded that prostate biopsy via mpMRI-TRUS fusion imaging can reduce diagnoses of clinically insignificant prostate cancers^[4,5]^ while achieving a comparable^[5]^ or even higher^[4]^ positive detection rate of clinically significant prostate cancers (csPCA), than 12-core systemic prostate biopsy.

Numerous studies have compared systemic transrectal biopsy and MR-GPB,^[5,6]^ whereas few studies have investigated the differences between MR-GPB and TTPSB. Due to its reliability in diagnosis, TTPSB, especially transperineal template–mapping biopsy, can be used not only as a diagnostic method for focal therapy of prostate cancer but also as a reference for the accuracy of MRI and transrectal biopsy.^[7]^ This study aims to investigate the accuracy of MR-GPB and TTPSB and compare the efficacy of detection rates for any cancer and csPCA and the spatial distribution of missed lesions by these 2 methods to provide evidence for clinical practice.

## 2 Materials and methods

### 2.1 Study population

This study was approved by the ethics committee of Peking Union Medical College Hospital, Chinese Academy of Medical Sciences. All patients provided informed consent.

A total of 240 consecutive patients were prospectively enrolled, and TTPSB was performed from May 2014 to June 2017. Patients satisfying all the following criteria were included in our study: age > 18 years old. PSA > 4 ng/mL, digital rectal examination (+), or hypoechoic nodule (s) on a prostate ultrasound test. No history of transurethral resection of the prostate, endocrine therapy for prostate cancer or previous prostate biopsyhistory. No contradictions for mpMRI.

A total of 226 patients underwent mpMRI, performed with a 3T scanner (GE HD750) without an endorectal coil before an eventual prostate biopsy.

### 2.2 mpMRI examination

**Figure 1:**
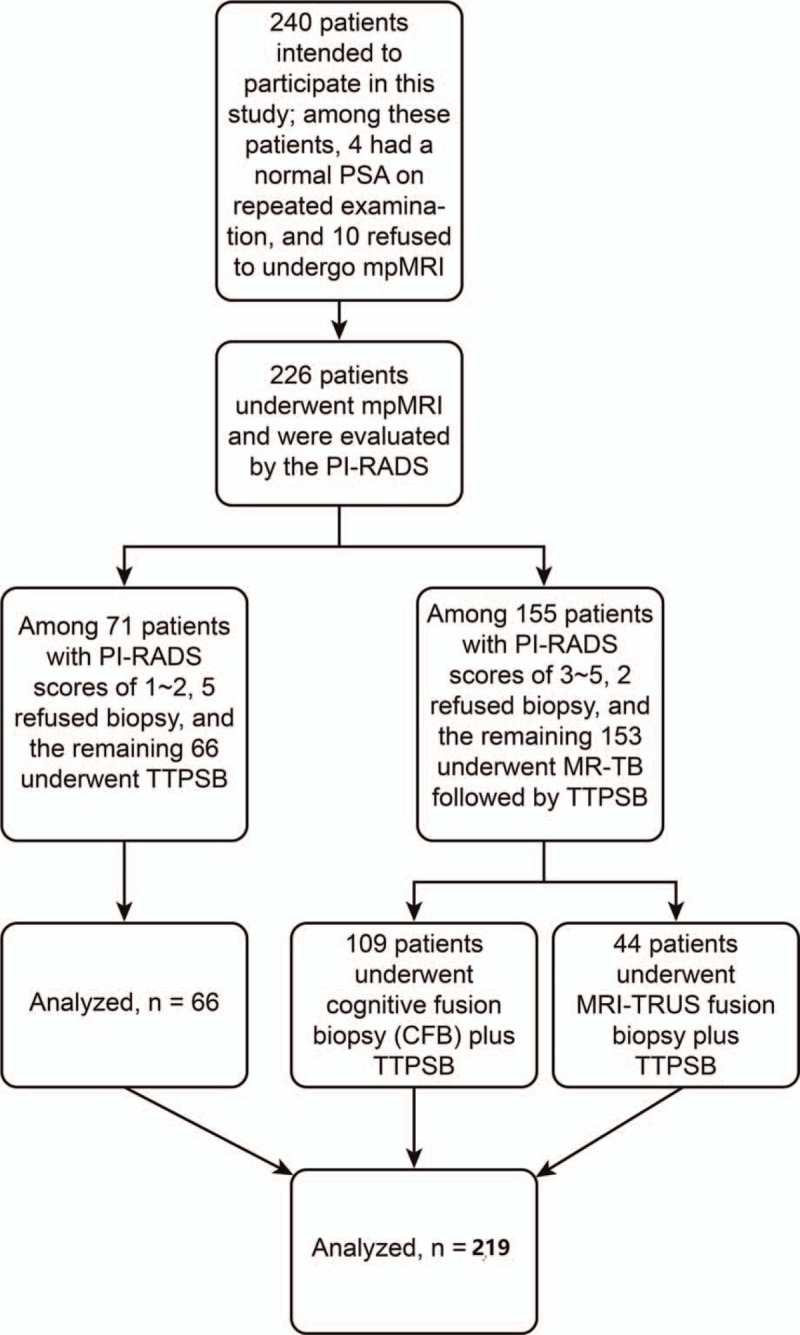
A flowchart of the study design of this research.

All reports were completed by one radiologist who had over 10 years of experience in reading prostate MRI images. The mpMRI scanning included T1-weighted, T2-weighted, diffusion-weighted with apparent diffusion coefficient, and dynamic contrast-enhanced sequences. The PI-RADS score was used to evaluate the possibility of a lesion being prostate cancer, and scores ranged from 1 to 5 indicating: highly unlikely, unlikely, equivocal, likely, and highly likely. In this study, 155 patients had a PI-RADS score of 3 to 5, 2 of whom rejected prostate biopsy. The remaining 153 patients underwent MR-GPB for suspected lesions (109 patients underwent cognitive fusion biopsy plus TTPSB, and 44 patients underwent MRI-TRUS fusion biopsy plus TTPSB). Seventy-one patients had a PI-RADS score of 1 to 2, 5of whom rejected biopsy, and the remaining 66 patients underwent TTPSB alone. A total of 219 patients were included in this study (Fig. 1).

### 2.3 Prostate biopsy procedure

All participants were inpatients of the Urology Department of Peking Union Medical College Hospital. For MRI-TRUS fusion biopsy, the radiologist, along with a urologist, located and delineated the lesions using mpMRI before surgery. The median time from mpMRI to biopsy was 11 days (5–19 days). All patients received intravenous general anesthesia in a lithotomy position and then underwent template-guided transperineal MR-GPB. Subsequently, another urologist who was blinded to the MRI results performed TTPSB on the same patient. We used a Hi Vision Prelrus ultrasound machine with a transrectal transducer (Hitachi, Tokyo, Japan) and a computer-assisted elastic image fusion system with real-time virtual sonography 3D tracking technology (Hitachi, Tokyo, Japan). Cognitive fusion biopsy patients also underwent MR-GPB first, and TTPSB was performed by another urologist who was blinded to the MRI results. TTPSB was performed in 11 regions.^[8]^The Bard Biopsy gun (C.R. Bard, Inc., Covington, GA) and an 18-gauge biopsy needle were used to obtain biopsy cores transperineally through the template.

csPCA was defined as a lesion with a Gleason score ≥3 + 4 or with a Gleason score ≥3 + 3 and a maximum cancer core length of 5 mm or larger.^[5]^

### 2.4 Study aim

The primary outcome of this study is the detection rate of MR-GPB and TTPSB for all prostate cancers and csPCA. Secondary outcomes include the distribution of missed cancer and the frequency of Gleason score upgrading by these 2 methods.

### 2.5 Statistical analysis

Data that were normally distributed, such as age, PSA and maximum cancer core length, are expressed as the mean± standard deviation. Other data, such as prostate volume, biopsy cores and PSA value are expressed as the median and interquartile range (IQR). McNemar's tests were used to compare detection rates between MR-GPB and TTPSB. SPSS 20.0 (Chicago, IL) was used to analyze the data. A 2-tailed value of *P < *.05 was considered statistically significant.

## 3 Results

### 3.1 Baseline data

The mean age of the 219 patients in the study was 64.3± 8.3 years old (ranging from 37 to 81 years old). The prostate-specific antigen (PSA) range was 0.03–48 ng/mL, with a median of 9.5 ng/mL (IQR: 6.5–15.5ng/mL). The prostate volume varied from 15 to 130 ml, with a median of 45 ml (IQR: 30–55 ml). Sixty-six patients had a PI-RADS score of 1 to 2 on mpMRI and underwent TTPSB alone, while 153 patients had a PI-RADS score of 3 to 5 and underwent TTPSB after MR-GPB. The median number of biopsy cores for patients who underwent MR-GPB was 2 (IQR: 2–5), whereas the median positive core was 1 (IQR: 1–2). The median number of biopsy cores of the 219 TTPSB patients was 23 (IQR: 20–28) in 11 areas, with 1 to 4 cores in one region. The median number of biopsy-positive regions was 4 (IQR 2–6). The mean maximal cancer core length was 0.6± 0.3 cm.

### 3.2 Detection rate

Of these 219 patients, 107 were diagnosed with prostate cancer, with a positive rate of 48.9% (107/219). Among the 107 patients with prostate cancer, a total of 94 patients were diagnosed with csPCA, with a detection rate of 42.9% (94/219). Detection rates for any cancer (TTPSB 87/219, 39.7%; MR-GPB 76/219, 34.7%; *P = *.161) and csPCA (TTPSB76/219, 34.7%; MR-GPB 72/219; 32.9%, *P = *.636) did not significantly differ between the 2 groups.

**Figure 2:**
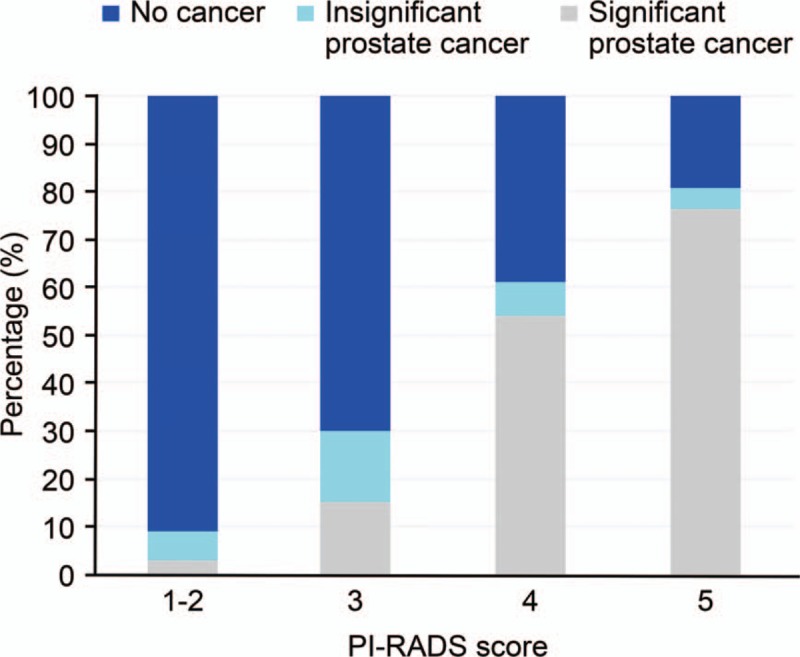
Percentage of men with clinically significant prostate cancer, clinically insignificant prostate cancer, and no cancer diagnosed by MRI-targeted biopsy and template-guided transperineal prostate saturation biopsy (TTPSB) within different PI-RADS score groups. MRI = magnetic resonance imaging, PI-RADS = Prostate Imaging Reporting and Data System, TTPSB = template-guided transperineal prostate saturation biopsy.

Sixty-six (30.1%) patients had normal findings on MRI (with a PI-RADS score of 1–2), and 9.1% (6/66) of these were positive upon biopsy, with 2 patients eventually diagnosed with csPCA. Additionally, 153 (69.9%) patients had a PI-RADS score of 3–5, 66.0% (101/153) of whom were positive on biopsy, and 60.1% (92/153) had csPCA. Among patients with a PI-RADS score of 4 to 5, 71.4% (95/133) were positive upon biopsy, whereas 66.9% (89/133) had csPCA. The efficacy of prostate cancer and csPCA detection in patients with different PI-RADS scores is listed in Figure 2.

**Figure 3:**
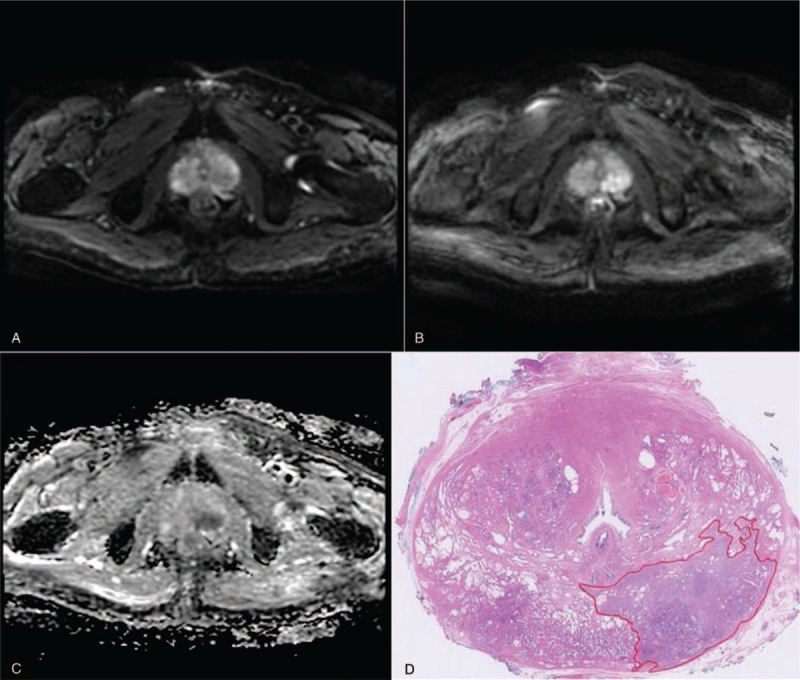
Results for a 72 years old man with prostate specific antigen of 8.7 ng/mL. Magnetic resonance imaging (MRI) suggested left posterior prostate cancer focus visible on (A) T2-weighted image, (B) diffusion weighted imaging, and (C) apparent diffusion coefficient map, the PI-RADS score was 5. A 3-core magnetic resonance-guided prostate biopsy and 11-region template-guided transperineal prostate saturation biopsy revealed Gleason 4 + 3 prostate cancer in left posterior prostate. The patient was treated with radical prostatectomy. (D) Whole-mount prostate section confirmed pT2 Gleason 4 + 3 prostate cancer. The tumor location corresponded to the location of positive cores and MRI. MRI = magnetic resonance imaging, PI-RADS = Prostate Imaging Reporting and Data System.

The prostate cancer focus in MRI and corresponding pathology are shown in Figure 3.

### 3.3 The spatial distribution of missed csPCA

Of 94 cases of csPCA, 23.4% (22/94) were missed by MR-GPB. However, TTPSB missed 19.1% (18/94) of csPCA cases (*P = *.476). csPCA lesions missed by TTPSB were most commonly located in the right anterior (3.2%, 3/94) and left anterior regions (4.3%, 4/94), and 6 of these 7 patients had a prostate volume greater than 60 mL. csPCA lesions missed by MR-GPB were most commonly located on the left (8.5%, 8/94) and right (9.6%, 9/94) sides of the urethra.

### 3.4 Gleason score

The median Gleason score was 7 (IQR: 6–8). Of the 66 patients with normal MRI findings, 2 had a Gleason score of 3 + 4, while 4 had a score of 3 + 3. Among patients diagnosed with prostate cancer, upgrading of the Gleason score occurred in 22.4% (24/107) who were diagnosed by MR-GPB and 25.2% (27/107) who were diagnosed by TTPSB (*P = *.630) (Table 1).

**Table 1:**
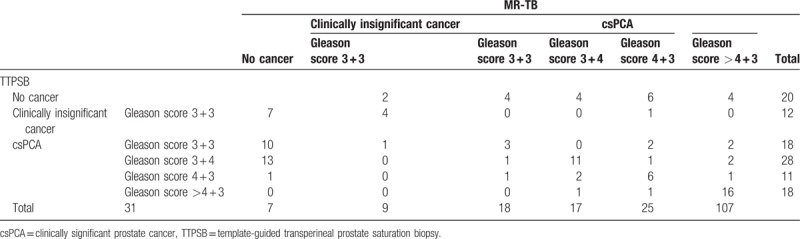
Pathological outcomes of prostate cancer biopsies by MR-TB and TTPSB.

## 4 Discussion

Based on our results, mpMRI is a sensitive method for detection of csPCA. Less than 10% of patients with negative MRI findings were eventually diagnosed with prostate cancer, 2/3 of whom had clinically insignificant prostate cancer. mpMRI can reduce unnecessary biopsies by 30%, with a false negative rate of only 5.6% (6/107) for prostate cancer and 2.1% (2/94) for csPCA, which is consistent with a previous report.^[7]^ These results indicate that MRI may potentially play a significant role in screening for prostate cancer, even though it is relatively expensive. Several studies claim that MR-GPB does not increase the efficacy of detection of high-grade prostate cancer in biopsy-naive men.^[9,10]^ However, for patients with a prior negative biopsy who are considered at higher risk, MR-GPB is becoming a standard csPCA detection method instead of repeated systematic biopsy.^[11,12]^

Our study shows that MR-GPB has great value for csPCA detection. Even with fewer cores, MR-GPB achieved an efficacy comparable to TTPSB. Baco et al^[5]^ reported that 2-core mpMRI-TRUS fusion biopsy had an efficacy equivalent to 12-core systemic TRUS-guided biopsy, whereas another study conducted by Siddiqui concluded that mpMRI-TRUS integrated target biopsy surpassed systemic biopsy in csPCA detection.^[4]^

Prostate lesions are graded from 1 to 5 by MRI findings to indicate the likelihood of being prostate cancer, and a score of 3 to 5 implies significant abnormality. In this study, we found that among patients with a PI-RADS score≥3, 60.8% (93/153) had csPCA, which is consistent with the results of Baco's study (66%).^[5]^ For lesions with a PI-RADS score of 4 to 5, the positive rate of csPCA was 66.9% (89/133), which was far lower than the value in Baco's report (97%).^[5]^ and comparable to that reported by Ahmed et al.^[7]^ However, it should be noted that Ahmed et al^[7]^ used a stricter definition of csPCA; therefore, a relatively low positive rate of csPCA would have been identified if we had adopted a similar definition. This difference might be associated with racial factors or inconsistencies in PI-RADS and pathological diagnostic standards. Ahmed et al reported that approximately 50% of the patients with PI-RADS scores of 1 to 2 had prostate cancer, and approximately 10% had csPCA, which is much higher than our results.

Generally, prostate cancer occurs multifocally.^[13]^ Different lesions or distinct parts of a same lesion might exhibit varied Gleason scores, thus different biopsy methods could lead to discrepant Gleason scores when different puncture sites are used.^[6,14]^ The results of MR-GPB and TTPSB could be combined as a “gold standard” because either MR-GPB or TTPSB alone exhibited a likelihood of 1/5–1/4 that the Gleason score would be upgraded, as shown in our study. This result suggests that these 2 methods share similar accuracy in Gleason scoring and further assures that MR-GPB with fewer cores can achieve an efficacy equivalent to TTPSB with >20 cores.

A missed prostate cancer diagnosis via biopsy is a common occurrence,^[1,15]^and various methods are prone to miss cancer lesions at different sites. For example, transrectal prostate biopsy misses prostate cancer in the anterior and apex of the prostate.^[1,2]^ According to our study, compared to MR-GPB, TTPSB tended to miss prostate cancer lesions in front of the urethra, especially in prostates with a larger volume. These misses might have occurred because larger prostates may be shielded by the pubic arch.^[8]^ To avoid this, we did not use the template and punctured obliquely upward in patients with pubic arch occlusion,^[8]^ which however, lowered the accuracy. The efficacy of biopsy in this area would be enhanced with MRI. It should be noted that MR-GPB is less sensitive for detection of cancer lesions in the transitional zones on both sides of the urethra, which emphasizes the need for competent ability in reading MRI images to reduce missed diagnoses.

Currently, 3 types of MR-GPB are available: in-bore MRI target biopsy, MRI-TRUS fusion biopsy, and cognitive fusion biopsy. These 3 methods have similar detection rates for csPCA. In addition, MRI-TRUS fusion biopsy and cognitive fusion biopsy are not significantly different in terms of overall prostate cancer detection.^[16]^Therefore, our MR-GPB group included 2 methods of biopsy: MRI-TRUS fusion biopsy and cognitive fusion biopsy. MRI-TRUS fusion biopsy is superior to cognitive fusion biopsy in detecting smaller prostate cancer lesions.^[17]^

Our study has some limitations. First, the MR-GPB group included 2 methods of biopsy: MRI-TRUS fusion biopsy and cognitive fusion biopsy. Nevertheless, some evidence shows that no significant differences in efficacy exist between these methods.^[16,17]^ Second, while the most reliable method to reveal missed diagnoses of cancer lesions is examining radical prostatectomy specimens, we did not collect these materials in our study. Third, we performed TTPSB after MR-GPB in patients with abnormal MRI findings. Thus, MR-GPB potentially affected ultrasound imaging of local hemorrhage and further impaired the efficacy of TTPSB, which was an inevitable systemic error. Fourth, though MR-GPB has a comparable efficacy to TTPSB for any cancer and csPCA detection, TTPSB may selected for diagnosing prostate cancer for its cost was cheaper than that of MR-GPB.

In conclusion, MR-GPB can avoid approximately 30% of unnecessary prostate biopsies and has a comparable efficacy to TTPSB for any cancer and csPCA detection. MR-GPB missed diagnoses of approximately 1/4 of csPCAs, which were most commonly located on both sides of the urethra.

## Author contributions

**Conceptualization:** Zhien Zhou, Zhiyong Liang, Zhigang Ji.

**Data curation:** Yi Zhou, Zhien Zhou, Yinyan Xu, Hao Sun, Zhiyong Liang, Wei-Gang Yan, Hanzhong Li.

**Formal analysis:** Qianyue Li, Yinyan Xu, Hao Sun, Yu Xiao, Wei-Gang Yan.

**Investigation:** Yinyan Xu.

**Methodology:** Yi Zhou, Zhien Zhou, Hao Sun, Zhiyong Liang, Wei-Gang Yan, Zhigang Ji, Hanzhong Li.

**Resources:** Wei-Gang Yan, Zhigang Ji.

**Software:** Hao Sun.

**Validation:** Hao Sun, Yu Xiao, Zhiyong Liang.

**Visualization:** Hao Sun.

**Writing – original draft:** Yi Zhou, Zhien Zhou, Yu Xiao, Wei-Gang Yan.

**Writing – review & editing:** Yi Zhou, Zhien Zhou, Qianyue Li, Wei-Gang Yan, Zhigang Ji.
